# Cost-effectiveness of cadonilimab plus chemotherapy vs chemotherapy alone for advanced gastric cancer: evidence to inform drug pricing in the U.S. and China

**DOI:** 10.3389/fimmu.2025.1618726

**Published:** 2025-10-14

**Authors:** Wenwang Lang, Liuyong Mei, Qiang Xiao, Zujin Zhou, Huiqing Jiang, Xianling Zhao

**Affiliations:** ^1^ Department of Pharmacy, Nanxishan Hospital of Guangxi Zhuang Autonomous Region, Guilin, China; ^2^ Department of Oncology, Nanxishan Hospital of Guangxi Zhuang Autonomous Region, Guilin, China; ^3^ Department of Spine Surgery, Nanxishan Hospital of Guangxi Zhuang Autonomous Region, Guilin, China

**Keywords:** cost-effectiveness, cadonilimab, PD-1/CTLA-4, gastric cancer, Markov model

## Abstract

**Background:**

Cadonilimab, a bispecific antibody targeting programmed cell death protein 1 (PD-1) and cytotoxic T-lymphocyte-associated protein 4 (CTLA-4), was the first agent of its class to demonstrate promising therapeutic efficacy in combination with chemotherapy for patients diagnosed with advanced gastric or gastroesophageal junction adenocarcinoma (GC/GEJC). This economic evaluation aimed to determine whether cadonilimab plus chemotherapy offers cost-effective benefits compared to chemotherapy alone from both the U.S. and Chinese healthcare payer perspectives. In addition, we estimated the pricing thresholds at which cadonilimab would be considered economically viable as a first-line treatment.

**Methods:**

We constructed a Markov model comprising three health states, progression-free survival (PFS), progressive disease (PD), and death, spanning a 10-year time horizon. The clinical efficacy data were sourced from the randomized phase 3 COMPASSION-15 trial. The cost and utility parameters were derived from existing literature. The model calculates total costs, quality-adjusted life-years (QALYs), and incremental cost-effectiveness ratios (ICERs). Subgroup, scenario, and sensitivity analyses were performed, and price simulations explored cost-effective thresholds at defined willingness-to-pay (WTP) levels.

**Results:**

In the base-case analysis, the cadonilimab plus chemotherapy provided an incremental gain of 0.33 QALYs at an additional cost of $16,797.61, resulting in an ICER of $50,582.10 per QALY, above the WTP threshold of China of $40,354.27 per QALY. In the U.S. setting, although the combination therapy achieved a slightly higher incremental QALY gain of 0.35 QALYs, the substantial additional cost of $101,275.06 resulted in an unfavorable ICER of $290,498.45 per QALY, exceeding the U.S. WTP threshold of $150,000.00. Among Chinese patients with a PD-L1 combined positive score (CPS) ≥5, the ICER was lower at $37,499.27/QALY, rendering the therapy cost-effective. Simulations identified cadonilimab pricing below $209.54/125 mg (China) and $826.46/125 mg (the U.S.) as necessary for cost-effectiveness.

**Conclusion:**

Cadonilimab combined with chemotherapy may be cost-effective in Chinese patients with elevated PD-L1 expression. However, its broader use in other patient subgroups or countries requires significant price reductions. These findings provide important guidance for future reimbursements and pricing decisions.

## Introduction

Gastric cancer (GC), including tumors located at the gastroesophageal junction (GEJC), is the fourth most prevalent malignancy globally and is a major contributor to cancer-related mortality (1). In 2020, it accounted for approximately 1.1 million new cases and over 768,000 deaths worldwide (2). Epidemiological data reveal substantial regional disparities in disease burden: China reports roughly 358,700 new GC/GEJC cases and more than 260,400 annual deaths (3), while the United States reports around 30,300 new cases and over 10,780 deaths each year (4). A critical shared challenge in both regions is that most patients are diagnosed at an advanced stage, which severely restricts treatment options and undermines long-term prognosis. The most common histological type of gastric cancer is adenocarcinoma, with the majority being human epidermal growth factor receptor 2 (HER2)-negative (5, 6). Despite advances in medical technology, more than 50% of patients with gastric cancer present with metastatic and unresectable tumors at diagnosis. Anti-programmed cell death protein-1 (PD-1) and programmed death ligand 1 (PD-L1) inhibitors combined with chemotherapy have become the standard of care for first-line treatment of HER2-negative, unresectable locally advanced or metastatic gastric or gastroesophageal junction (GC/GEJ) adenocarcinoma (7–11). Although the addition of a PD-1 inhibitor to chemotherapy improves outcomes, survival benefits remain limited in patients with low PD-L1 expression.

Cadonilimab is a human tetravalent bispecific IgG1 antibody with a symmetric IgG single-chain variable fragment (scFv) structure and an Fc-null design to eliminate antibody-dependent cellular cytotoxicity (ADCC), antibody-dependent cellular phagocytosis (ADCP), complement-dependent cytotoxicity (CDC), and cytokine release. Fc receptor-mediated effector functions can eliminate or impair lymphocytes expressing PD-1 and cytotoxic T-lymphocyte-associated protein 4 (CTLA-4), thereby reducing their antitumor activity (**Supplementary Figure 1**). Moreover, immune-related adverse events (irAEs) induced by checkpoint inhibitors have been associated with the recruitment of immune cells bearing Fc receptors (12, 13). Cadonilimab has shown promising clinical activity and manageable safety in patients with gastric or GEJ adenocarcinoma, regardless of PD-L1 expression (14).

In the phase 3 COMPASSION-15 trial (15), both progression-free survival (PFS) and overall survival (OS) significantly improved in the cadonilimab group. In the intention-to-treat (ITT) population, the median OS was 15.0 months versus 10.8 months; in patients with PD-L1 combined positive score (CPS) ≥5, the median OS was not reached versus 10.6 months; and in those with PD-L1 CPS <5, it was 14.8 months versus 11.1 months. The median PFS was 7.0 months in the cadonilimab group compared to 5.3 months in the placebo group. Among patients with PD-L1 CPS ≥5, PFS was 6.9 months with cadonilimab and 4.6 months with placebo, while in the PD-L1 CPS <5 group, PFS was 6.9 months versus 5.5 months.

Cadonilimab is the world’s first PD-1/CTLA-4 bispecific antibody tumor immunotherapy drug developed independently in China and provides critical evidence supporting updates to clinical practice guidelines for gastric cancer. Despite its clinical potential, there is no comprehensive evidence of its economic value. Cadonilimab has been granted orphan drug status and fast-track designation by the U.S. FDA and is expected to receive market approval as early as 2026. As a next-generation immunotherapy agent, it is projected to be a key component of the global oncology market, which is valued at nearly USD 100 billion. However, its high price triggered two rounds of price reductions in China, from $1,856.30 to $865.80, and then to $261.17 per 125 mg, raising concerns about affordability and cost-effectiveness. The absence of pricing information in the U.S. further complicates economic evaluations. Additionally, cadonilimab may soon be included in National Comprehensive Cancer Network (NCCN) Guidelines (16), underscoring the need for cost-effectiveness data to inform clinical and policy decisions in both regions.

This study aimed to assess the cost-effectiveness of cadonilimab in combination with chemotherapy versus chemotherapy alone as first-line treatment for advanced GC/GEJC from both U.S. and Chinese healthcare payer perspectives, thereby informing future drug pricing and reimbursement decisions.

## Methods

### Patient enrollment and intervention

This study followed the Consolidated Health Economic Evaluation Reporting Standards (CHEERS) guidelines (17). Eligible patients were between 18 and 75 years of age and had histologically confirmed, locally advanced, unresectable, or metastatic GC/GEJC. None of the patients had previously received systemic therapy for advanced disease. Patient characteristics and inclusion criteria were consistent with those described in the COMPASSION-15 clinical trial.

The participants were randomly assigned to receive either cadonilimab (10 mg/kg, administered intravenously) or a placebo every 21 days for up to 24 months. Both groups received concurrent chemotherapy with capecitabine (1,000 mg/m² orally, twice daily on days 1–14) and oxaliplatin (130 mg/m² intravenously on day 1), repeated in 21-day cycles for up to six cycles (XELOX regimen). Following the combination phase, the patients continued with cadonilimab or placebo as monotherapy.

Subsequent treatments, including PD-1 inhibitors, targeted agents, chemotherapy, or best supportive care, were performed in accordance with the NCCN (16) and Chinese Society of Clinical Oncology (CSCO) guidelines for gastric cancer (18) and were consistent with post-treatment strategies used in the COMPASSION-15 trial (15). Tumor assessments were performed every six weeks during the first 54 weeks after enrollment and every nine weeks thereafter.

Adverse events (AEs) were monitored with a particular focus on severe (grade ≥3) events occurring in more than 3% of patients. These included anemia, neutropenia, thrombocytopenia, and hypokalemia (**Table 1**).

**Table 1:** Key clinical input data.

Parameters	Baseline value	Range		Distribution	Reference
		Minimum	Maximum		
*Survival model for OS*					
Cadonilimab plus chemotherapy	Meanlog=2.7363 Sdlog=0.9902			Lognormal	(15)
chemotherapy	Shape=2.0400 Scale=11.3080			Loglogistic	(15)
*Survival model for PFS*					
Cadonilimab plus chemotherapy	Mu=1.9580 Sigma=0.9908 Q=-0.4756			Generalized gamma	(15)
chemotherapy	Meanlog=1.6818 Sdlog=0.7658			Lognormal	(15)
*Survival model for OS (CPS ≥5)*					
Cadonilimab plus chemotherapy	Meanlog=2.8810 Sdlog=1.1670			Lognormal	(15)
chemotherapy	Shape=1.9017 Rate=0.1390			Gamma	(15)
*Survival model for PFS (CPS ≥5)*					
Cadonilimab plus chemotherapy	Meanlog=2.1741 Sdlog=0.9829			Lognormal	(15)
chemotherapy	Meanlog=1.6784 Sdlog=0.7388			Lognormal	(15)
*Survival model for OS (CPS <5)*					
Cadonilimab plus chemotherapy	Meanlog=2.6772 Sdlog=0.9669			Lognormal	(15)
chemotherapy	Meanlog=2.4639 Sdlog=0.8078			Lognormal	(15)
*Survival model for PFS (CPS <5)*					
Cadonilimab plus chemotherapy	Meanlog=2.0993 Sdlog=0.9096			Lognormal	(15)
chemotherapy	Meanlog=1.6956 Sdlog=0.7848			Lognormal	(15)
*Drug cost, $/per cycle*					
Cost of Cadonilimab	1305.87	1044.70	1567.04	Gamma	Local charge
Cost of Tislelizumab	352.03	281.62	422.44	Gamma	Local charge
Cost of Oxaliplatin	166.25	133.00	199.50	Gamma	Local charge
Cost of Capecitabine	44.06	35.25	52.87	Gamma	Local charge
Cost of 5-FU	126.87	101.50	152.24	Gamma	Local charge
Cost of Cisplatin	34.66	27.73	41.59	Gamma	Local charge
Cost of Paclitaxel	212.61	170.09	255.13	Gamma	Local charge
Cost of Ramucirumab	2106.24	1702.93	2554.39	Gamma	Local charge
Testing for PD-L1 protein biomarker	567.64	454.11	681.17	Gamma	Local charge
Cost of the laboratory test	106.61	85.29	127.93	Gamma	(19)
Enhanced CT	171.03	136.82	205.24	Gamma	Local charge
Cost of end-of-life	1460.30	1168.24	1752.36	Gamma	(20)
Best supportive care	164.57	92.16	138.24	Gamma	(20)
Cost of drug administration per unit	134.93	107.94	161.92	Gamma	(21, 22)
*Proportion of receiving subsequent treatment in Cadonilimab plus chemotherapy group*					
PD-L1/PD-1 Medication	11.50%	9.20%	13.80%	Beta	(15)
Chemotherapy Regimen	34.40%	27.52%	41.28%	Beta	(15)
Targeted therapy	11.10%	8.88%	13.32%	Beta	(15)
*Proportion of receiving subsequent treatment in Chemotherapy group*					
PD-L1/PD-1 Medication	22.30%	17.84%	26.76%	Beta	(15)
Chemotherapy Regimen	47.90%	38.32%	57.48%	Beta	(15)
Targeted therapy	20.30%	16.24%	24.36%	Beta	(15)
*Cost of AEs, $*					
Anemia	669.45	535.56	803.34	Gamma	(23)
Decreased platelet count	1054.22	843.38	1265.06	Gamma	(23)
Decreased neutrophil count	544.19	435.35	653.03	Gamma	(23)
Hypokalemia	3000.00	2400.00	3600.00	Gamma	(23)
*Utilities*					
Utility of PFS	0.797	0.638	0.956	Beta	(24)
Utility of PD	0.577	0.462	0.692	Beta	(24)
*Disutility estimates*					
Anemia	0.07	0.06	0.084	Beta	(23)
Decreased platelet count	0.11	0.09	0.132	Beta	(23)
Decreased neutrophil count	0.20	0.16	0.240	Beta	(23)
Hypokalemia	0.04	0.09	0.14	Beta	(23)
*Risk for main AEs in Cadonilimab plus chemotherapy group*					
Anemia	10.20%	8.16%	12.24%	Beta	(15)
Decreased platelet count	28.50%	22.80%	34.20%	Beta	(15)
Decreased neutrophil count	15.10%	12.08%	18.12%	Beta	(15)
Hypokalemia	5.90%	4.72%	7.08%	Beta	(15)
*Risk for main AEs in Chemotherapy group*					
Anemia	12.50%	10.00%	15.00%	Beta	(15)
Decreased platelet count	25.00%	20.00%	30.00%	Beta	(15)
Decreased neutrophil count	14.80%	11.84%	17.76%	Beta	(15)
Hypokalemia	1.00%	0.08%	0.12%	Beta	(15)
Discount rate	5%	4.00%	6.00%	Beta	
BMI/m2	1.72				
Weight/kg	65				
$1 = ¥7.0467	40,354.27				

OS, overall survival; PFS, progression-free survival; PD, progression disease; CPS, PD-L1 combined of positive score; AE, adverse event; BMI, body mass index.

### Model structure

**Figure 1:**
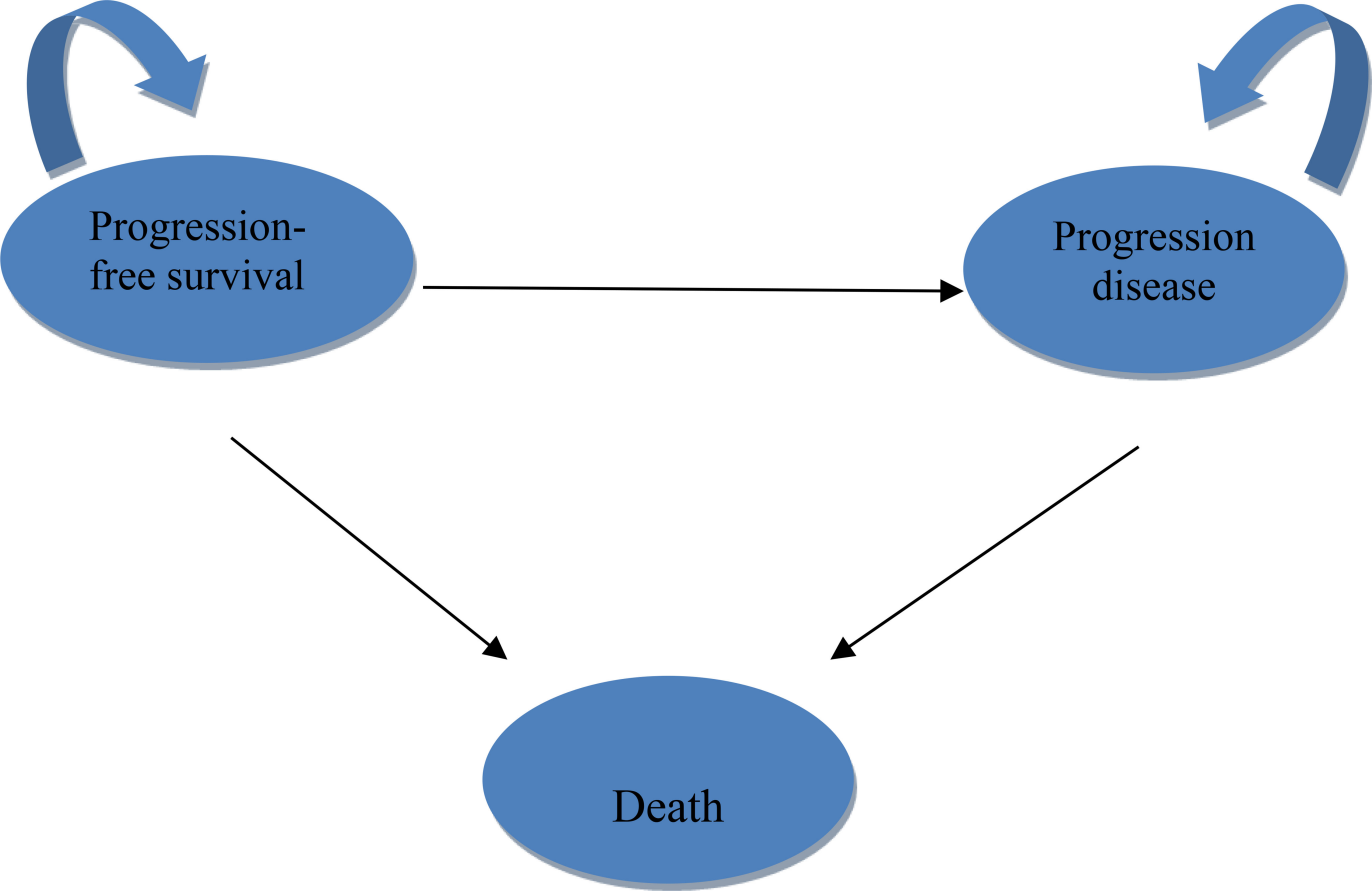
Markov model structure.

A three-state Markov model was constructed using TreeAge Pro 2022 (Williamstown, MA, USA) and R version 4.2.4 (Vienna, Austria). Health states included PFS, progressive disease (PD), and death (**Figure 1**). The model employed a 3-week cycle length over a 10-year time horizon, representing the lifetime of the patient population and capturing over 99% mortality.

The analysis was conducted from the perspective of healthcare payers in both China and the United States. The Chinese model adopted a system-wide healthcare payer perspective, whereas the U.S. analysis focused on direct medical costs relevant to both public and private payers (25).

### Outcomes

The model evaluated total life years, quality-adjusted life years (QALYs), incremental cost-effectiveness ratios (ICERs), incremental net health benefits (INHB), and incremental net monetary benefits (INMB). Annual discount rates were applied to both costs and utilities—3% for the U.S. and 5% for China—in accordance with established pharmacoeconomic guidelines (26, 27).

Chinese cost data were converted to 2024 U.S. dollars using an exchange rate of $1 = ¥7.1217 and were adjusted for inflation using the local consumer price index. The willingness-to-pay (WTP) thresholds were set at $40,354.27 per QALY in China (three times the national gross domestic product per capita) and $150,000 per QALY in the U.S., consistent with standards established by the WHO and U.S. healthcare payers (28).

### Clinical data inputs

Probabilities for OS and PFS were extracted from Kaplan–Meier (KM) curves in the ASTRUM-005 trial using the GetData Graph Digitizer (http://getdata-graph-digitizer.com), and individual patient data were reconstructed following the method described by Guyot et al. (29).

Due to limited follow-up, extrapolation was required to extend survival estimates across the model’s full time horizon (30). The reconstructed time-to-event data were then fitted with a series of parametric models, including classic models (exponential, Weibull, Gompertz, gamma, log-logistic, log-normal, and generalized gamma).

**Figure 2:**
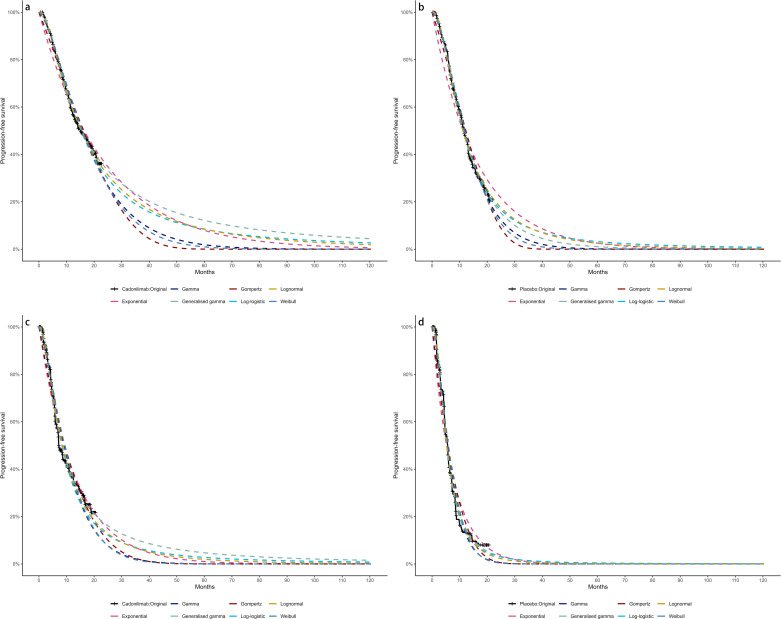
The Kaplan-Meier: **(A)** overall survival curves of Cadonilimab plus chemotherapy group, **(B)** overall survival curves of Chemotherapy group, **(C)** progression-free survival curves of Cadonilimab plus chemotherapy group, **(D)** progression-free survival curves of Chemotherapy group.

**Figure 3:**
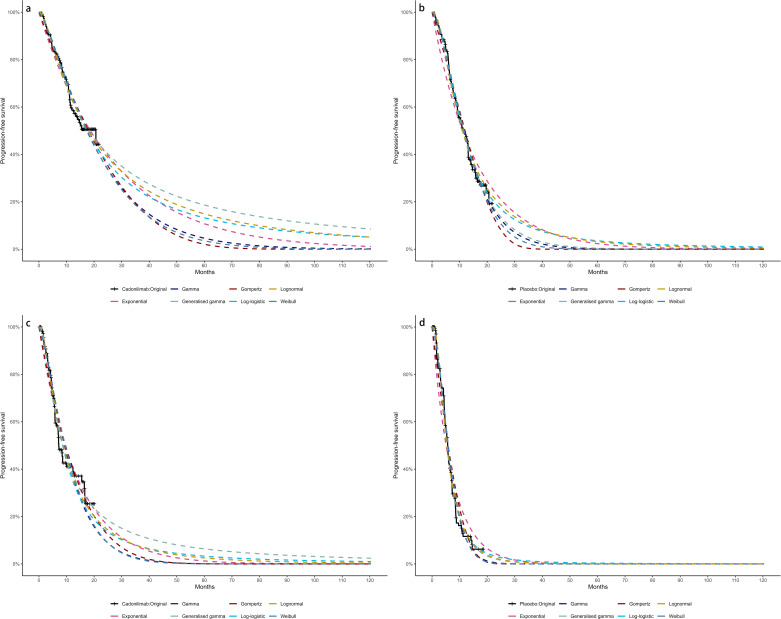
The Kaplan-Meier of CPS ≥ 5: **(A)** overall survival curves of Cadonilimab plus chemotherapy group, **(B)** overall survival curves of Chemotherapy group, **(C)** progression-free survival curves of Cadonilimab plus chemotherapy group, **(D)** progression-free survival curves of Chemotherapy group.

**Figure 4:**
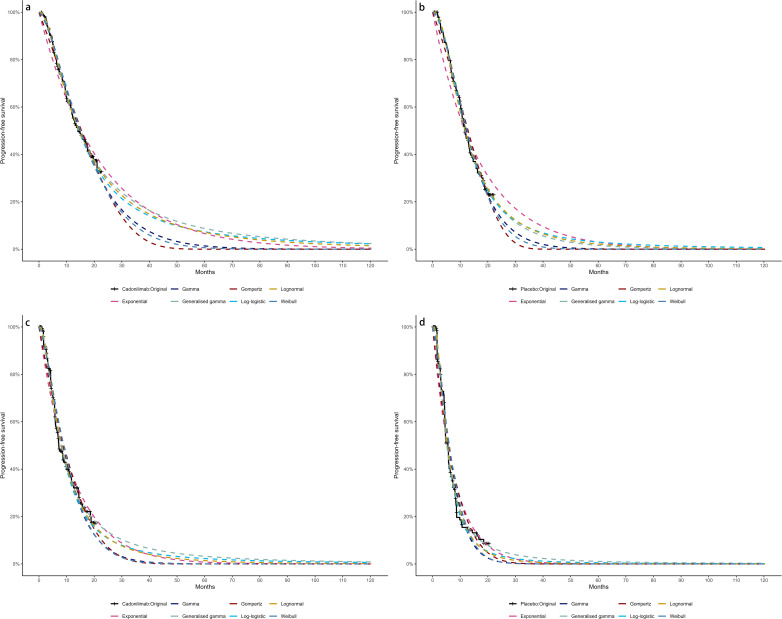
The Kaplan-Meier of CPS <5: **(A)** overall survival curves of Cadonilimab plus chemotherapy group, **(B)** overall survival curves of Chemotherapy group, **(C)** progression-free survival curves of Cadonilimab plus chemotherapy group, **(D)** progression-free survival curves of Chemotherapy group.

**Figure 5:**
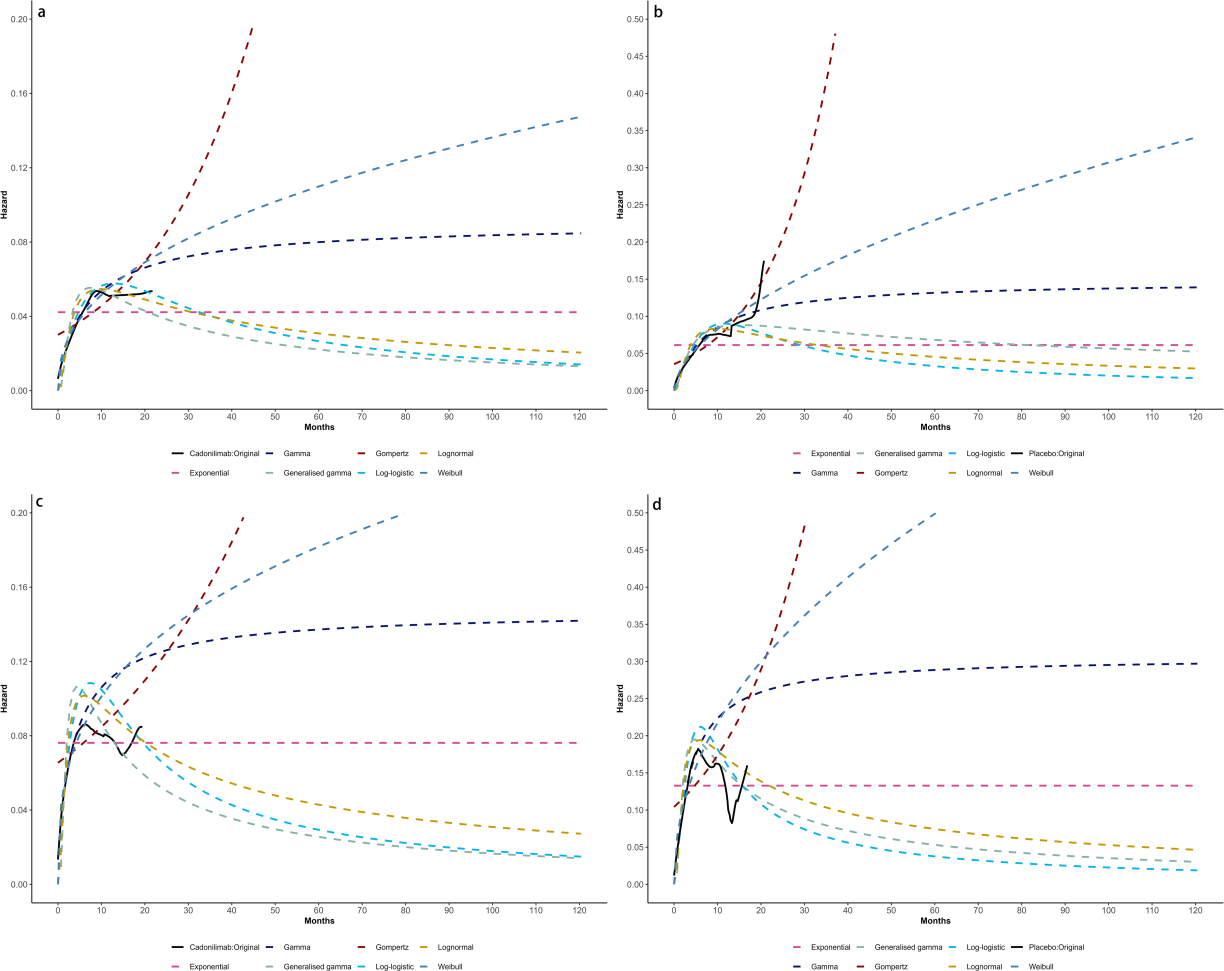
Comparison of fitted proportional hazards survival models with observed kaplan–meier curves: **(A)** overall survival curves of Cadonilimab plus chemotherapy group, **(B)** overall survival curves of Chemotherapy group, **(C)** progression-free survival curves of Cadonilimab plus chemotherapy group, **(D)** progression-free survival curves of Chemotherapy group.

**Figure 6:**
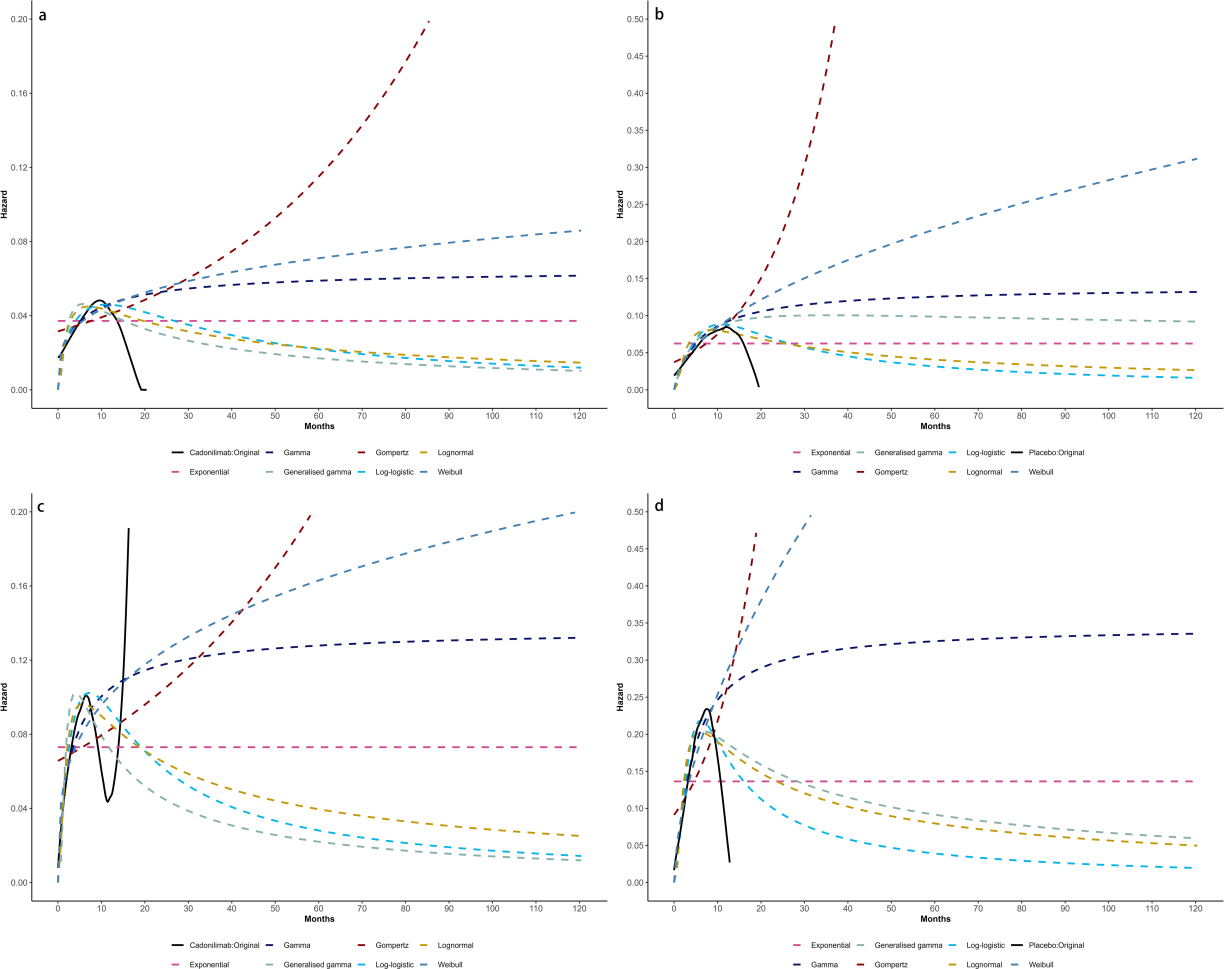
Comparison of fitted proportional hazards survival models with observed kaplan–meier curves of CPS ≥ 5: **(A)** overall survival curves of Cadonilimab plus chemotherapy group, **(B)** overall survival curves of Chemotherapy group, **(C)** progression-free survival curves of Cadonilimab plus chemotherapy group, **(D)** progression-free survival curves of Chemotherapy group.

**Figure 7:**
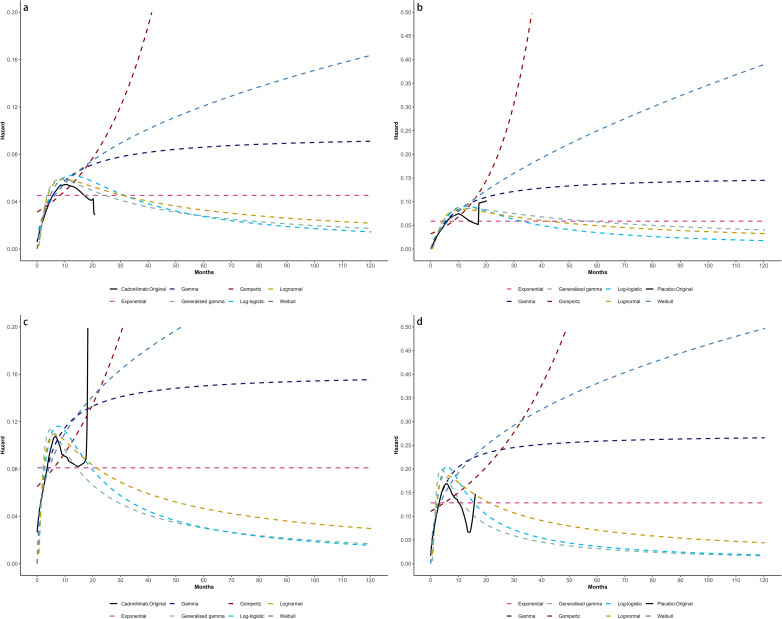
Comparison of fitted proportional hazards survival models with observed kaplan–meier curves of CPS <5: **(A)** overall survival curves of Cadonilimab plus chemotherapy group, **(B)** overall survival curves of Chemotherapy group, **(C)** progression-free survival curves of Cadonilimab plus chemotherapy group, **(D)** progression-free survival curves of Chemotherapy group.

Model selection was guided by a combination of statistical goodness-of-fit based on the Akaike information criterion (AIC), extrapolation performance based on log likelihood (LogLik), and visual inspection. Using this framework, the most suitable parametric model was chosen to extrapolate KM curves for OS and PFS beyond the follow-up period of the COMPASSION-15 trial (consistent with the trial referenced earlier for treatment protocols) (**Figures 2**–**4**). Before implementing the Cox proportional hazards (PH) model, the PH assumption—a core prerequisite for valid model inference—was validated using two complementary methods: visual inspection of log-log survival curves and quantitative assessment of Schoenfeld residuals (31, 32). While the PH assumption yielded p-values > 0.05 for both OS and PFS across all patient groups (nominally suggesting the assumption was satisfied), two critical observations indicated potential violation: crossing cumulative hazard curves between the treatment and control arms, and a non-horizontal trend in the smoothed Schoenfeld residuals. The variation in predicted hazards across different parameter distributions is shown in **Figures 5**–**7**. The corresponding survival function parameters are detailed in **Tables 2**–**4**.

**Table 2:** The Akaike information criteria (AIC), Bayesian information criteria (BIC) and Log likelihood (LogLik).

Type of distribution	Cadonilimab plus chemotherapy (OS)			Chemotherapy (OS)			Cadonilimab plus chemotherapy (PFS)			Chemotherapy(PFS)		
	AIC	BIC	LogLik	AIC	BIC	LogLik	AIC	BIC	LogLik	AIC	BIC	LogLik
Exponential	1284.687	1288.407	-641.344	1488.312	1492.032	-743.156	1195.999	1199.719	-596.999	1360.303	1364.023	-679.151
Gamma	1260.438	1267.878	-628.219	1438.741	1446.182	-717.371	1171.728	1179.169	-583.864	1298.264	1305.705	-647.132
Generalized gamma	1252.248	1263.409	-623.124	1438.235	1449.395	-716.117	1152.010	1163.171	-573.005	1278.456	1289.617	-636.228
Gompertz	1278.451	1285.892	-637.226	1463.253	1470.693	-729.626	1195.445	1202.886	-595.722	1351.569	1359.010	-673.785
Weibull	1264.650	1272.091	-630.325	1443.448	1450.888	-719.724	1179.156	1186.597	-587.578	1314.639	1322.080	-655.319
Log-logistic	1256.508	1263.949	-626.254	1437.368	1444.808	-716.684	1158.399	1165.839	-577.199	1279.473	1286.913	-637.736
Lognormal	1251.234	1258.674	-623.617	1437.727	1445.168	-716.863	1152.9990	1160.440	-574.499	1277.656	1285.097	-636.828

OS, overall survival; PFS, progression-free survival; AIC, Akaike information criterion; BIC, Bayesian information criterion; LogLik, Log likelihood.

**Table 3:** The Akaike information criteria (AIC), Bayesian information criteria (BIC) and Log likelihood (LogLik) (CPS ≥5).

Type of distribution	Cadonilimab plus chemotherapy (OS)			Chemotherapy(OS)			Cadonilimab plus chemotherapy (PFS)			Chemotherapy (PFS)		
	AIC	BIC	LogLik	AIC	BIC	LogLik	AIC	BIC	LogLik	AIC	BIC	LogLik
Exponential	431.287	434.040	-214.620	673.620	676.562	-335.810	428.832	431.585	-213.416	630.366	633.307	-314.183
Gamma	429.017	434.525	-212.477	655.971	661.855	-325.986	422.720	428.227	-209.360	595.904	601.788	-295.952
Generalized gamma	427.851	436.112	-210.906	657.836	666.661	-325.918	416.104	424.365	-205.052	593.860	602.685	-293.930
Gompertz	432.654	438.161	-214.296	664.185	670.068	-330.092	430.376	435.884	-213.188	620.211	626.095	-308.106
Weibull	429.856	435.363	-212.895	657.056	662.939	-326.528	425.200	430.707	-210.600	601.944	607.828	-298.972
Log-logistic	427.599	433.106	-211.774	656.027	661.911	-326.014	417.733	423.240	-206.866	592.798	598.681	-294.399
Lognormal	426.077	431.584	-211.016	658.737	664.621	-327.369	415.505	421.012	-205.752	591.928	597.811	-293.964

OS, overall survival; PFS, progression-free survival; AIC, Akaike information criterion; BIC, Bayesian information criterion; LogLik, Log likelihood; CPS, PD-L1 combined of positive score.

**Table 4:** The Akaike information criteria (AIC), Bayesian information criteria (BIC) and Log likelihood (LogLik) (CPS <5).

Type of distribution	Cadonilimab plus chemotherapy (OS)			Chemotherapy (OS)			Cadonilimab plus chemotherapy (PFS)			Chemotherapy (PFS)		
	AIC	BIC	LogLik	AIC	BIC	LogLik	AIC	BIC	LogLik	AIC	BIC	LogLik
Exponential	698.054	701.110	-348.027	723.001	725.991	-360.500	648.459	651.515	-323.229	655.088	658.078	-326.544
Gamma	684.856	690.969	-340.428	695.367	701.348	-345.683	634.040	640.152	-315.020	631.682	637.663	-313.841
Generalized gamma	682.361	691.529	-338.180	695.279	704.250	-344.639	626.171	635.340	-310.086	614.320	623.291	-304.160
Gompertz	694.703	700.816	-345.352	709.257	715.238	-352.629	647.587	653.699	-321.793	655.037	661.018	-325.519
Weibull	687.161	693.274	-341.581	698.274	704.255	-347.137	638.087	644.200	-317.044	640.021	646.002	-321.793
Log-logistic	682.887	688.999	-339.443	694.586	700.567	-345.293	628.307	634.419	-312.153	618.165	624.146	-307.082
Lognormal	680.510	686.623	-338.255	693.371	699.352	-344.685	625.246	631.359	-310.623	616.300	622.280	-306.150

OS, overall survival; PFS, progression-free survival; AIC, Akaike information criterion; BIC, Bayesian information criterion; LogLik, Log likelihood; CPS, PD-L1 combined of positive score.

### Cost inputs

This analysis focused exclusively on the direct medical costs associated with the management of GC/GEJC. These costs include drug acquisition, laboratory testing, enhanced computed tomography (CT), intravenous drug administration, subsequent therapies, best supportive care, end-of-life care, and management of severe adverse events (grade 3 or 4). Medication prices were obtained from public Chinese databases and institutional pricing schedules, whereas other cost components were derived from published economic evaluations and relevant literature.

Owing to the absence of a listed market price for cadonilimab in the United States, its cost was estimated using a comparative approach. Specifically, pricing was approximated based on analogous immunotherapies such as toripalimab and tislelizumab (33). Drug prices for both China and the U.S. were converted to U.S. dollars and adjusted using a price index to ensure cross-national comparability. **Tables 1** and **5** summarize the clinical and cost parameters used in this analysis (19–23, 28, 34, 35).

**Table 5:** Key clinical input data (US).

Parameters	Baseline value	Range		Distribution	Reference
		Minimum	Maximum		
*Drug cost, $/per cycle*					
Cost of Cadonilimab	8640.00	6912.00	10368.00	Gamma	Estimated
Cost of Pembrolizumab	11520.60	9216.48	13824.72	Gamma	(34)
Cost of Oxaliplatin	40.95	31.45	47.17	Gamma	(34)
Cost of Capecitabine	56.00	87.92	131.88	Gamma	(34)
Cost of 5-FU	50.17	40.14	60.20	Gamma	(34)
Cost of Cisplatin	42.32	36.63	54.95	Gamma	(34)
Cost of Paclitaxel	40.06	32.36	48.54	Gamma	(34)
Cost of Ramucirumab	13124.36	10499.49	15749.23	Gamma	(34)
Cost of the laboratory test	111.65	89.32	133.98	Gamma	(34)
Enhanced CT	424.35	339.48	509.22	Gamma	(34)
Testing for PD-L1 protein biomarker	459.00	367.20	550.80	Gamma	(34)
Cost of end-of-life	21603.00	17282.40	25923.60	Gamma	(28)
Best supportive care	3049.00	2439.20	3658.80	Gamma	(28)
Cost of drug administration first hour	142.55	114.04	171.06	Gamma	(34)
Administration intravenous, additional hour	30.68	24.54	36.82	Gamma	(34)
Cost of AEs, $					
Anemia	20260.00	6352.80	9529.20	Gamma	(35)
Decreased platelet count	22698.00	10484.00	15726.00	Gamma	(35)
Decreased neutrophil count	17181.00	10484.00	15726.00	Gamma	(35)
Hypokalemia	25326.00	6705.75	10058.63	Gamma	(35)
Discount rate	3%	0.02	0.04	Beta	
BMI/m2	2.1				
Weight/kg	75				

AE, adverse event; BMI, body mass index.

### Quality-of-life inputs

Health outcomes in the model were adjusted using utility values obtained from previously published sources, as EQ-5D-5L (European Quality of Life-5 Dimension-5 Level) data were not directly reported in the COMPASSION-15 trial. Utility values were anchored on a scale ranging from 0 (representing death) to 1 (representing perfect health).

For patients in the PFS state, the utility was set at 0.797 based on data from the TOGA trial and calculated using the Japanese EuroQol (EQ-5D) scoring algorithm (24). The utility for patients in the PD state was 0.577, derived from evaluations conducted by the National Institute for Health and Clinical Excellence (NICE). Quality-of-life decrements (disutilities) associated with severe adverse events, including anemia, thrombocytopenia, neutropenia, and hypokalemia, were also incorporated into the model (24). All AEs were assumed to occur during the initial treatment cycle, with detailed incidence rates provided in **Table 1**.

### Subgroup analyses

To explore heterogeneity in cost-effectiveness outcomes, subgroup analyses were performed for patients with PD-L1 CPS ≥5 and CPS <5 in both China and the United States. These analyses employed the same modeling structure and assumptions as those used in the base-case scenario. Due to the lack of subgroup-specific data on follow-up treatments, adverse event rates, or healthcare resource utilization in the COMPASSION-15 trial, these parameters were assumed to be consistent with those observed in the overall study population.

### Price simulation

Owing to uncertainties in the key input parameters, particularly drug pricing, scenario analyses were conducted to evaluate a range of potential pricing outcomes. In the Chinese context, cost-effectiveness was assessed with and without the inclusion of a patient assistance program. The price of cadonilimab varied between $0 and $2,000 per 625 mg dose, and outcomes were compared against the country-specific WTP threshold of $40,354.27 per QALY.

In the United States, where a formal list price for cadonilimab is currently unavailable, an estimated cost of $8,600 per 750 mg dose was used. This estimate was based on price comparisons with other anti-PD-1 agents, including toripalimab and tislelizumab. Scenario analyses in the U.S. setting varied the price from $0 to $10,000 per dose to identify the maximum price at which cadonilimab would remain cost-effective under a $150,000 WTP threshold.

### Scenario analysis

To address potential inconsistencies between the base-case discount rates (3% for the U.S., 5% for China) and World Health Organization (WHO) recommendations, an additional scenario was run where China’s discount rate was lowered to 3% (matching the U.S. rate). This analysis evaluated how aligning discount rates across regions would impact cost-effectiveness conclusions, particularly for long-term survival outcomes. Given the volatility of the yuan–dollar exchange rate in 2024–2025, a second scenario incorporated Purchasing Power Parity (PPP) adjustments to currency conversion—replacing the base-case market exchange rate with 2024 International Monetary Fund (IMF) PPP values (￥1 = $0.2825, equivalent to ￥3.54 = $1). Concurrently, China’s WTP threshold was adjusted to $20,243.85 per QALY to align with PPP-adjusted economic benchmarks, ensuring cross-country comparability of cost-effectiveness results under a standardized economic metric.

### Sensitivity analysis

The robustness of the model outcomes was evaluated using one-way sensitivity analysis (OWSA) and probabilistic sensitivity analysis (PSA). In the OWSA, each key parameter was independently varied by ±20% from its base-case value to determine its influence on the ICER. The results were visualized using tornado diagrams to identify the most influential variables.

For the PSA, all model inputs were sampled simultaneously based on appropriate probability distributions, beta for probabilities and utility values, and gamma for cost parameters. A total of 10,000 Monte Carlo simulations were performed to quantify the uncertainty in ICER estimates and to calculate the likelihood that cadonilimab plus chemotherapy would be deemed cost-effective at different WTP thresholds.

## Results

### Base-case analysis

Over a 10-year time horizon, the base-case analysis indicated that patients receiving cadonilimab in combination with chemotherapy achieved 1.01 QALYs at a total cost of $28,528.60. In contrast, those treated with chemotherapy alone accrued 0.67 QALYs at a cost of $11,730.98. This resulted in an incremental gain of 0.33 QALYs and an additional cost of $16,797.61, yielding an ICER of $5,0582.10 per QALY for the combination therapy (**Table 6**).

**Table 6:** The base case analysis.

Treatment	Cost	QALY	Incremental cost	Incremental QALY	INHB	INMB	ICER
Cadonilimab plus chemotherapy(China)	28528.60	1.01	16797.61	0.33	-0.08	-3396.52	50582.10
Chemotherapy(China)	11730.98	0.67					
Cadonilimab plus chemotherapy(CPS ≥5)(China)	32039.12	1.18	21999.87	0.59	0.04	1674.96	37499.27
Chemotherapy(CPS ≥5)(China)	10039.25	0.59					
Cadonilimab plus chemotherapy(CPS <5)(China)	27699.35	0.91	16349.88	0.25	-0.16	-6355.16	66013.60
Chemotherapy(CPS <5)(China)	11349.47	0.66					
Cadonilimab plus chemotherapy(US)	191469.60	1.04	101275.06	0.35	-0.33	-48981.29	290498.45
Chemotherapy(US)	90194.54	0.69					
Cadonilimab plus chemotherapy(CPS ≥5)(US)	225496.39	1.22	150226.89	0.62	-0.38	-56677.19	240877.66
Chemotherapy(CPS ≥5)(US)	75269.50	0.60					
Cadonilimab plus chemotherapy(CPS <5)(US)	190095.49	0.93	103745.44	0.26	-0.43	-64728.99	398852.61
Chemotherapy(CPS <5)(US)	86350.04	0.67					

QALY, Quality-adjusted life year; ICER, Incremental cost-effectiveness ratio; INMB, the incremental net monetary benefits; INHB, the incremental net health benefits; CPS, PD-L1 combined of positive score.

When compared to China’s WTP threshold of $40,354.27 per QALY, this ICER exceeded the acceptable limit. Consequently, the incremental net health benefit (INHB) was -0.08 QALYs, and the incremental net monetary benefit (INMB) was -$3,396.52, suggesting that cadonilimab plus chemotherapy is not cost-effective in the Chinese healthcare setting (**Table 6**).

In the U.S. scenario, the ICER for cadonilimab plus chemotherapy was estimated at $347,127.52 per QALY—well above the U.S. WTP threshold of $150,000.00. The corresponding INHB and INMB values were -0.41 QALYs and -$61,121.30, respectively, further supporting the conclusion that the combination regimen is not economically favorable under the current U.S. pricing assumptions (**Table 6**).

### Subgroup analysis

Among patients with a PD-L1 CPS ≥5, the ICER for cadonilimab plus chemotherapy was $37,499.27 per QALY—below China’s WTP threshold of $40,354.27 (**Table 6**). The corresponding INHB and INMB were 0.04 QALYs and $1,674.96, respectively, indicating that cadonilimab was cost-effective in this clinically responsive subgroup.

In contrast, for patients with a PD-L1 CPS <5, the ICER was $66,013.60 per QALY, exceeding the WTP threshold. The INHB was –0.16 QALYs and the INMB was –$6,355.16, suggesting that cadonilimab was not cost-effective in this lower PD-L1 expression group (**Table 6**).

In the U.S. setting, the ICER for cadonilimab plus chemotherapy reached $240,877.66 per QALY in the PD-L1 CPS ≥5 group and $398,852.61 per QALY in the CPS <5 group, both of which far exceeded the WTP threshold of $150,000.00 (**Table 6**). The corresponding INHBs were -0.38 and -0.43 QALYs, while the INMBs were -$56,677.19 and -$64,728.99, respectively. These findings indicate that cadonilimab was not cost-effective in either subgroup within the U.S. healthcare context, despite differential clinical responsiveness.

### Price simulation

**Figure 8:**
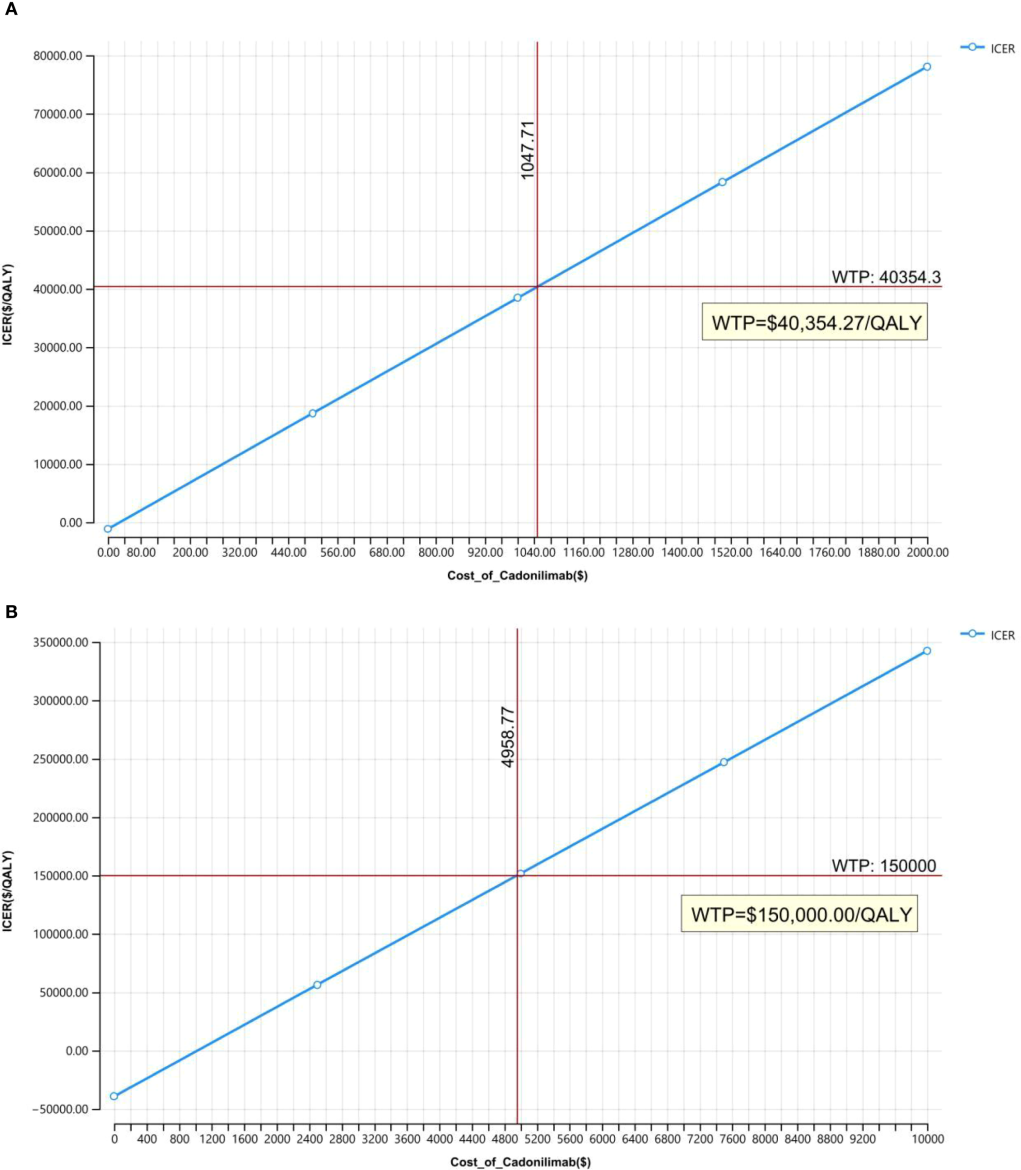
Price simulation: **(A)** China, **(B)** The U.S.

**Figure 8** illustrated the results of the price simulation analysis across a range of cadonilimab pricing scenarios. In China, the ICER increased proportionally as the price varied from $0 to $2,000 per 625 mg dose. A similar trend was observed in the United States, where the price range examined ranged from $0 to $10,000 per 750 mg dose.

According to the respective WTP thresholds, cadonilimab would be considered cost-effective in China if the price was below $209.54 per 125 mg. In the U.S., the threshold for cost-effectiveness was $826.46 per 125 mg.

### Scenario analysis

Scenario analysis evaluating a 3% discount rate for China showed the ICER of cadonilimab plus chemotherapy decreased to $48,678.70 per QALY, though the reduction was minimal (**Table 7**). For this scenario, PSA results indicated a 29.57% probability that the regimen would be cost-effective at China’s defined WTP threshold. Under PPP-adjusted currency conversion, the ICER of the combination regimen was 25,374.68 per QALY—still exceeding the PPP−aligned WTP of 20,243.85 per QALY (**Table 7**). PSA findings for this scenario showed a 24.86% probability of cost-effectiveness at the defined WTP threshold.

**Table 7:** Scenario analysis.

Treatment	Cost	QALY	Incremental cost	Incremental QALY	INHB	INMB	ICER
Cadonilimab plus chemotherapy(China)	29119.94	1.04	16970.62	0.35	-0.07	-2902.11	48678.70
Chemotherapy(China)	12149.32	0.69					
Cadonilimab plus chemotherapy(CPS ≥5)(China)	14311.46	1.01	8426.58	0.33	-0.08	-1703.88	25374.68
Chemotherapy(CPS ≥5)(China)	5884.89	0.67					

QALY, Quality-adjusted life year; ICER, Incremental cost-effectiveness ratio; INMB, the incremental net monetary benefits; INHB, the incremental net health benefits; CPS, PD-L1 combined of positive score.

### Sensitivity analysis

**Figure 9:**
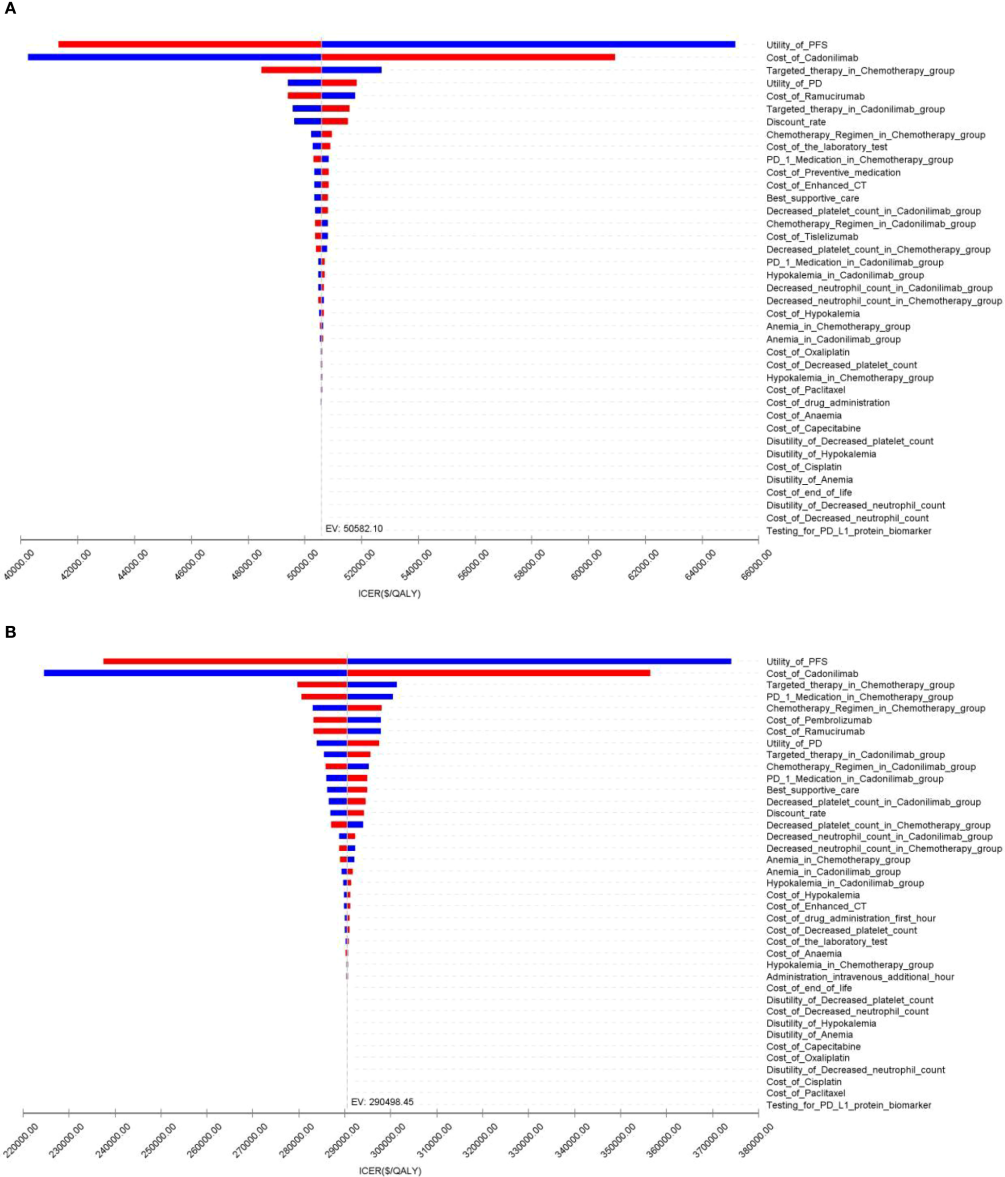
The tornado diagram of one-way sensitivity analysis: **(A)** China, **(B)** The U.S.

**Figure 10:**
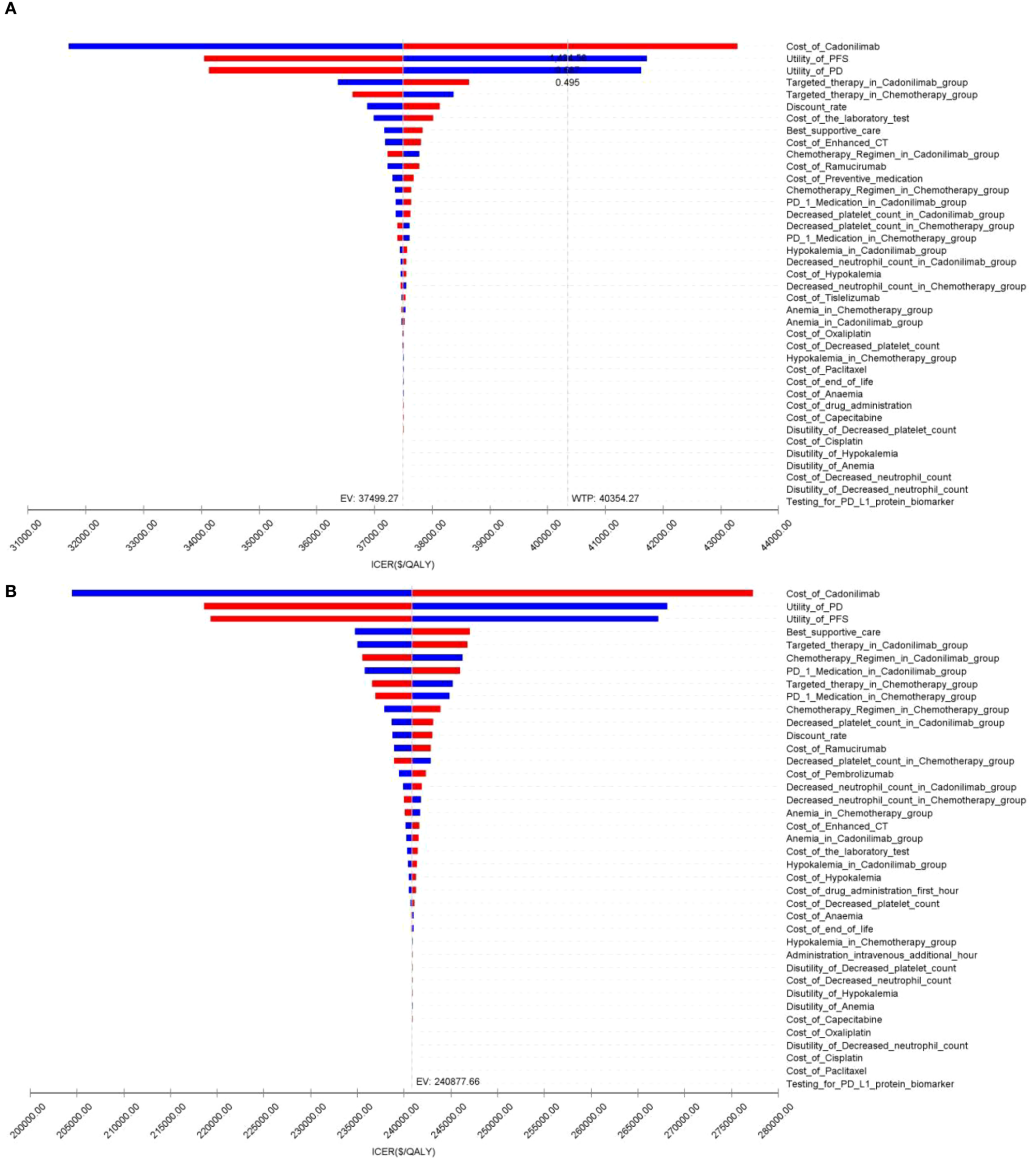
The tornado diagram of one-way sensitivity analysis in CPS ≥5 group: **(A)** China, **(B)** The U.S.

**Figure 11:**
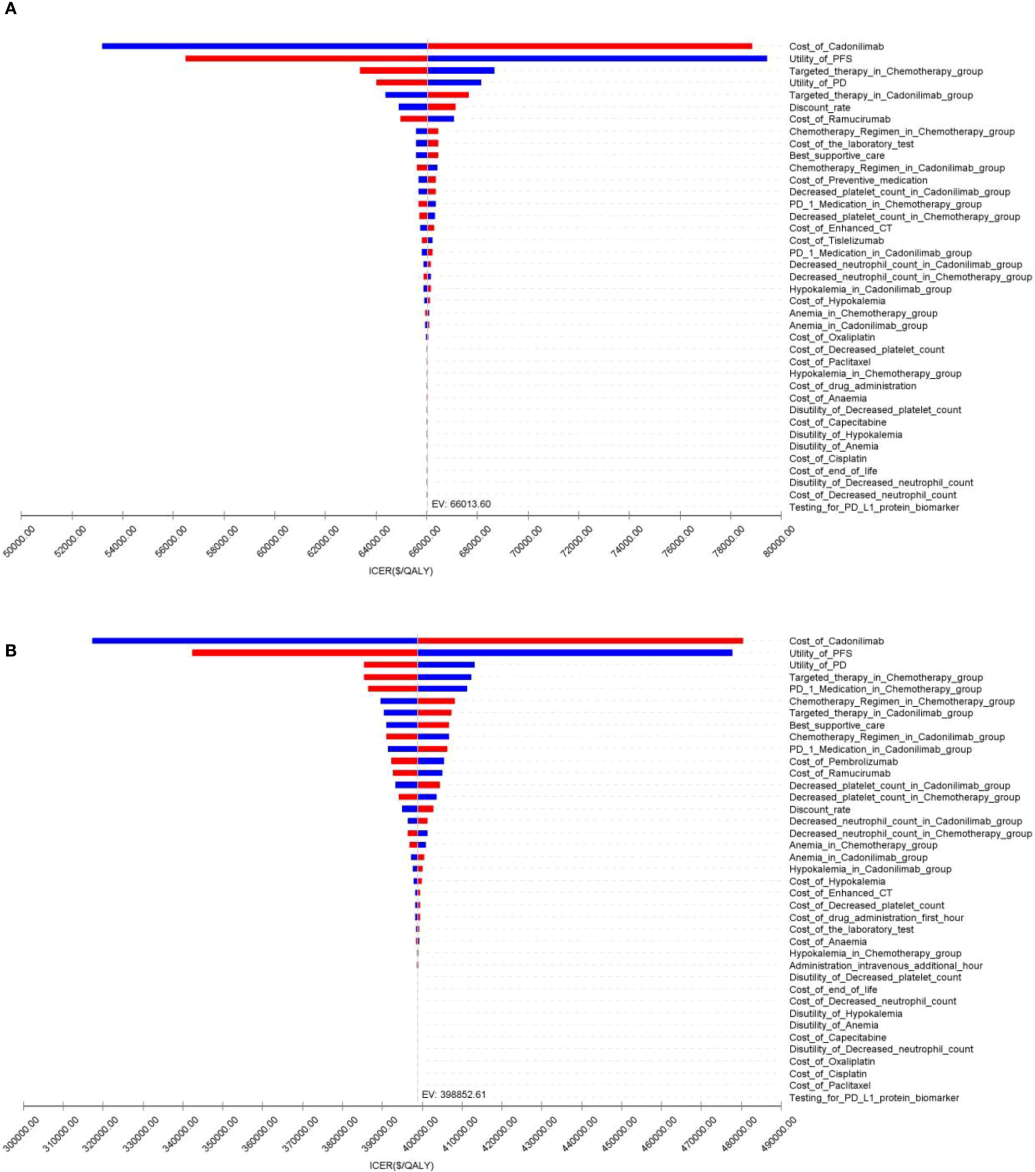
The tornado diagram of one-way sensitivity analysis in CPS <5 group: **(A)** China, **(B)** The U.S.

The OWSA results for the overall population and all subgroups in both China and the U.S. are presented in **Figures 9**–**11**. The ICER was most sensitive to variations in the cost of cadonilimab, utility values for the PFS and PD health states, and the proportion of patients receiving targeted therapy during subsequent treatment. Despite these sensitivities, the differences in health outcomes between treatment strategies were sufficiently large that parameter variations did not alter the overall conclusions, except in the CPS ≥5 subgroup in China, where the ICER was influenced by changes in drug cost and health state utilities.

**Figure 12:**
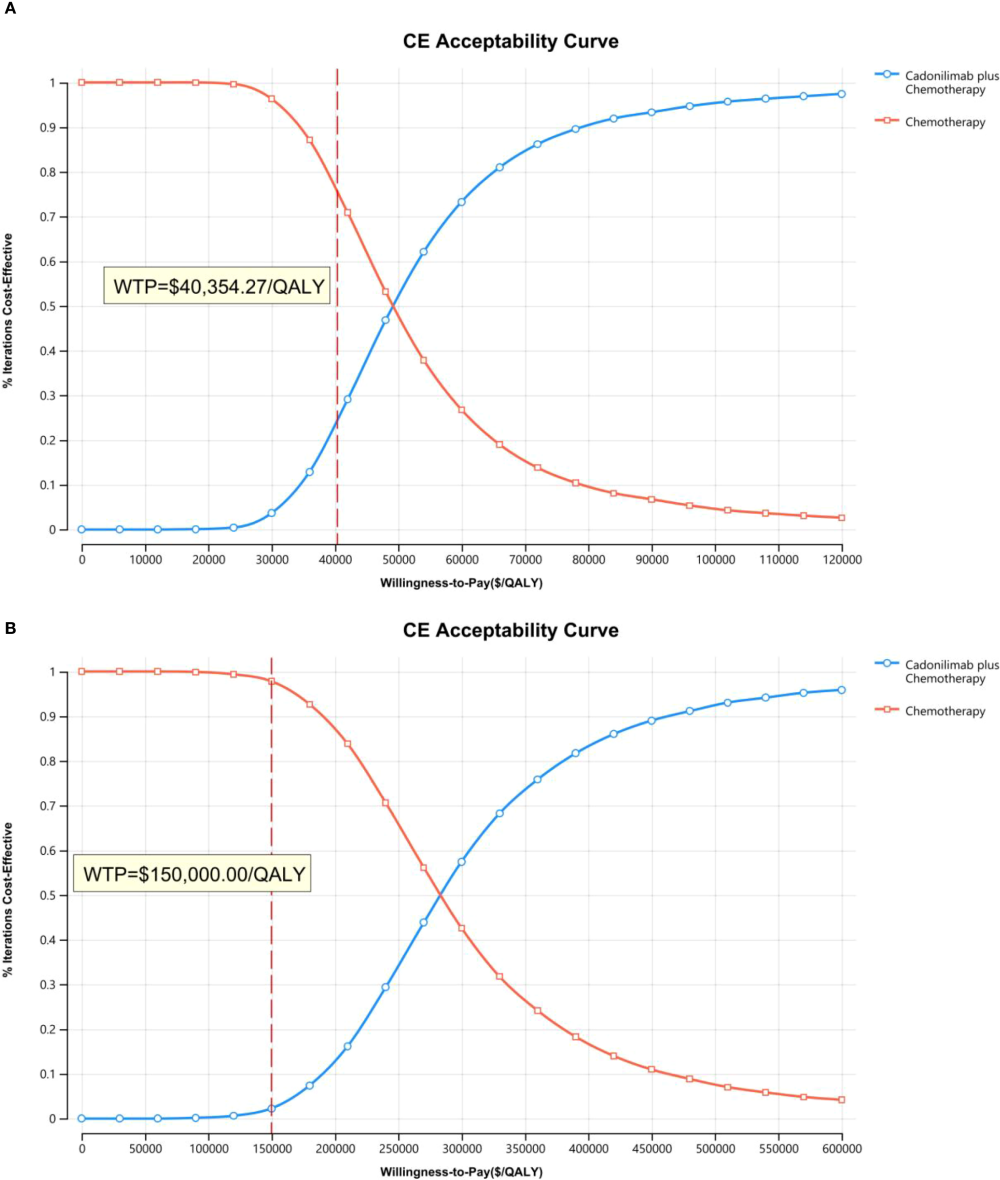
The cost-effectiveness acceptability curve: **(A)** China, **(B)** The U.S.

**Figure 13:**
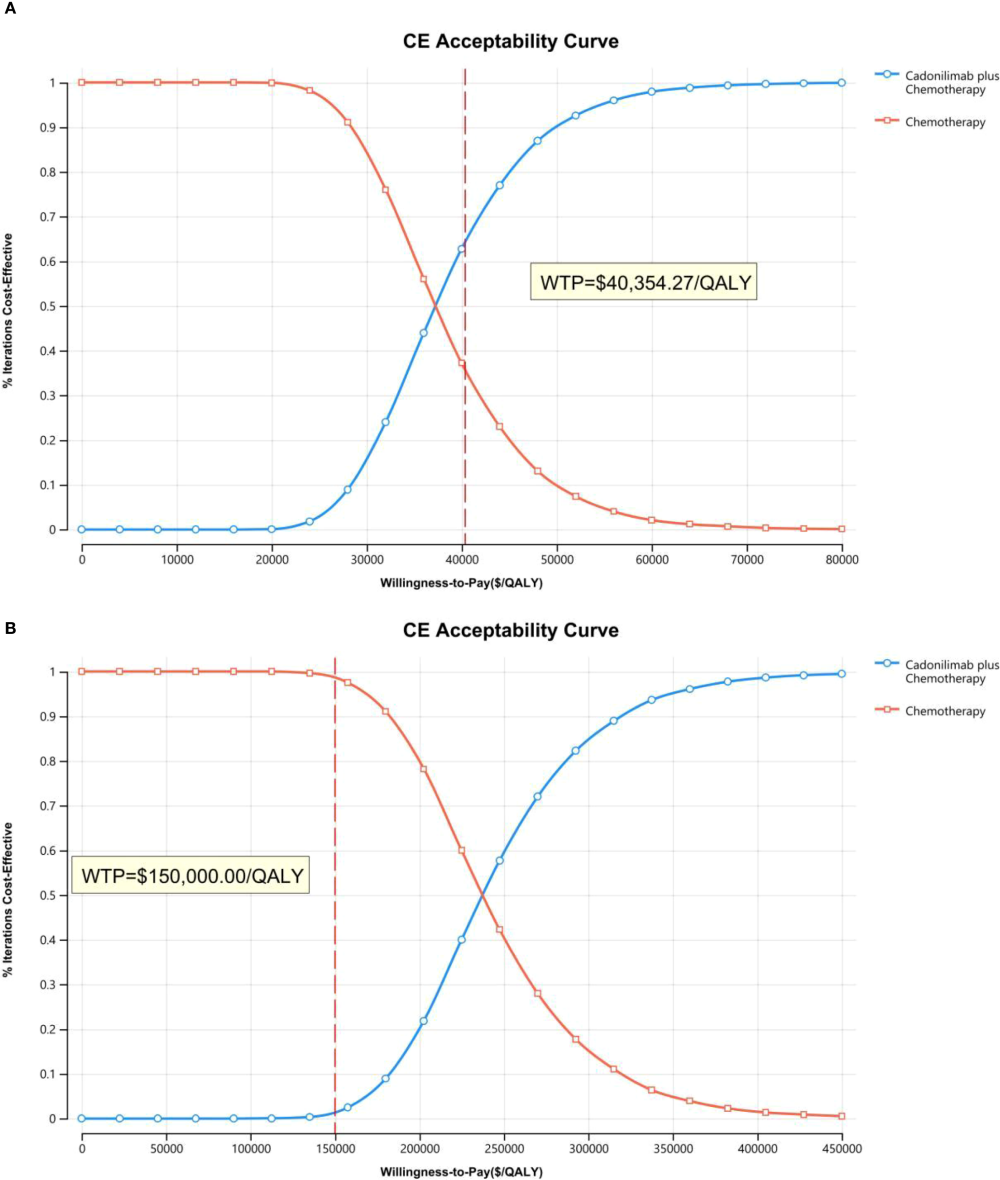
The cost-effectiveness acceptability curve in CPS ≥5 group: **(A)** China, **(B)** The U.S.

**Figure 14:**
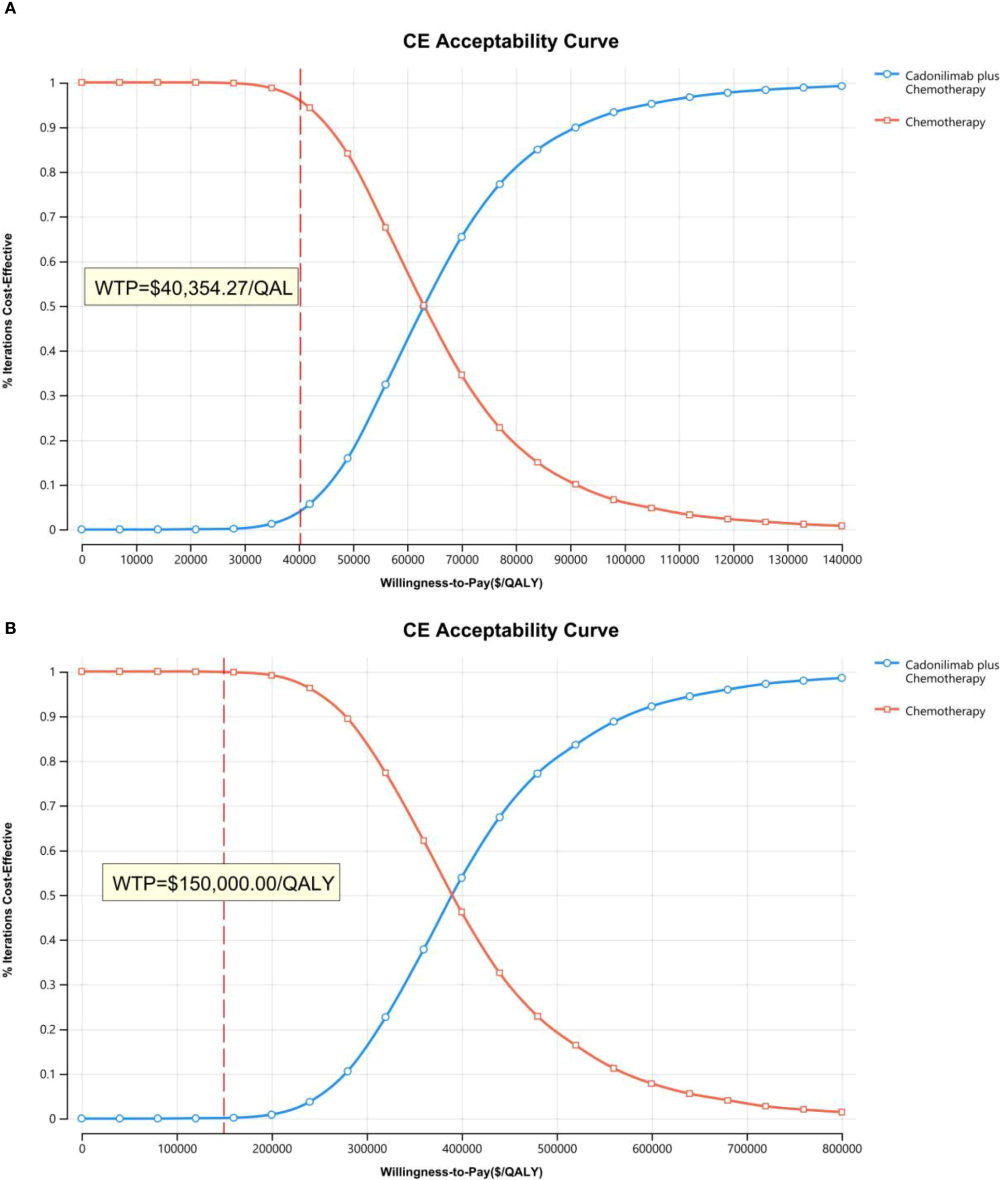
The cost-effectiveness acceptability curve in CPS <5 group: **(A)** China, **(B)** The U.S.

**Figure 15:**
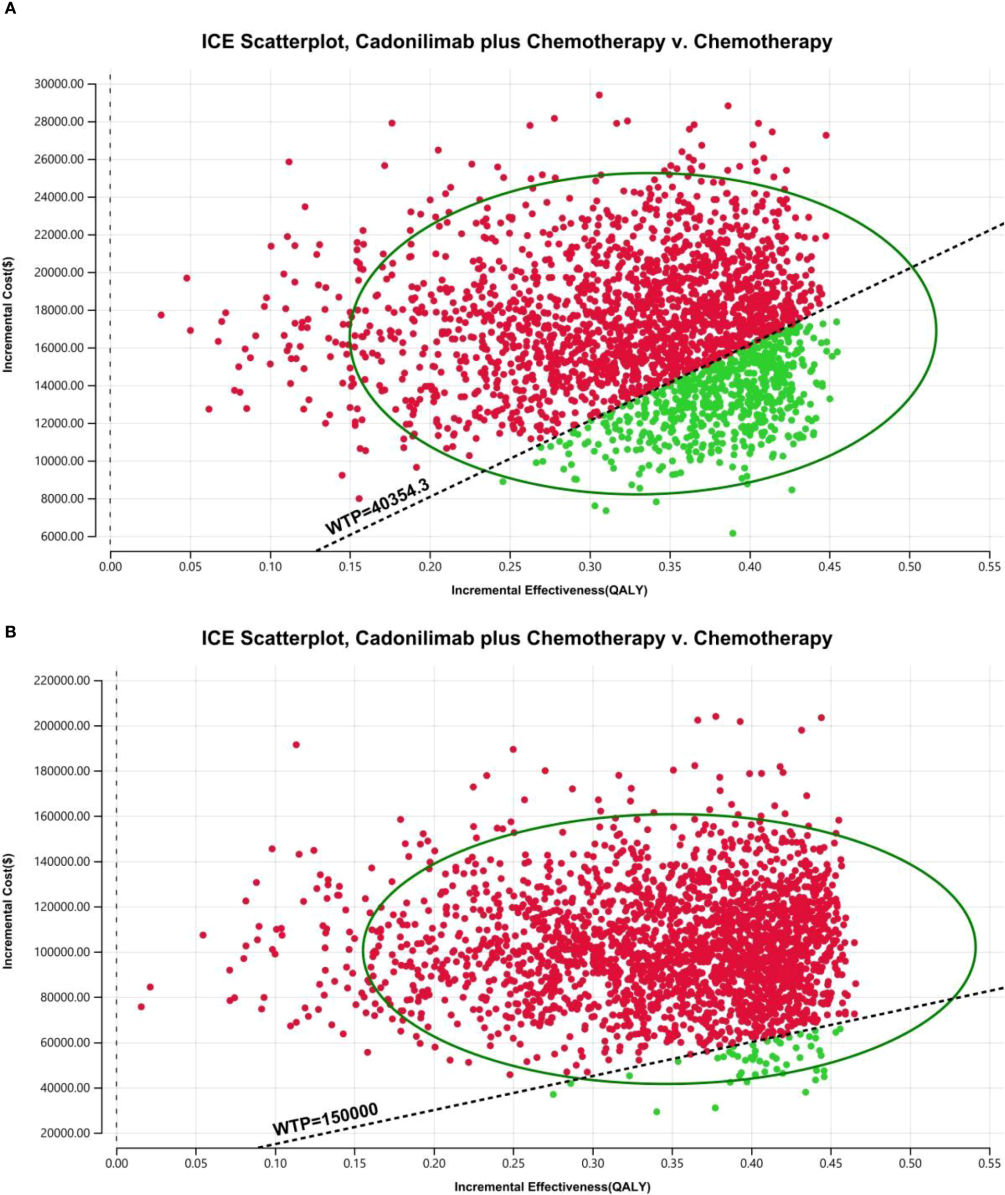
The cost-effectiveness probabilistic scatter plot: **(A)** China, **(B)** The U.S.

**Figure 16:**
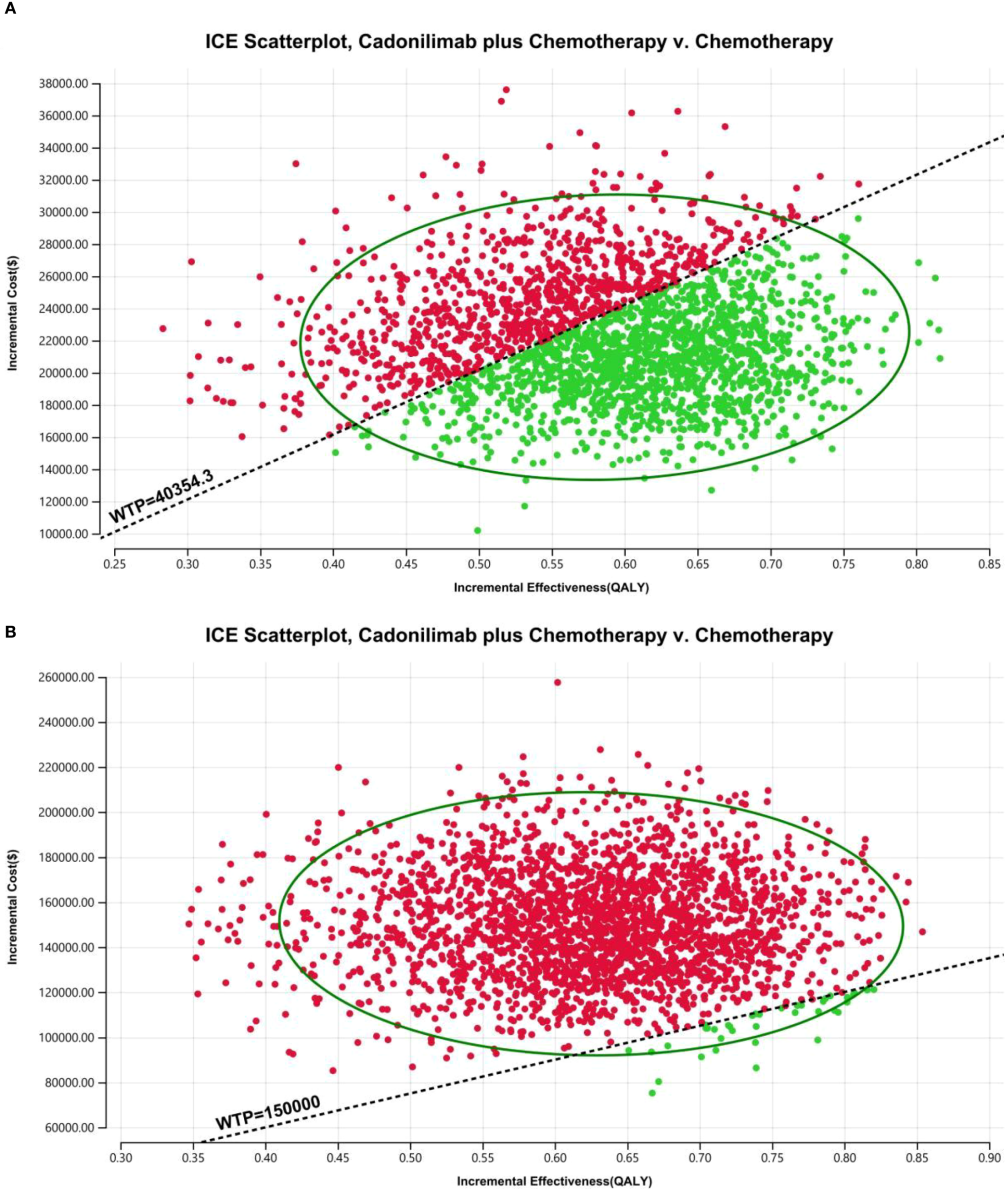
The cost-effectiveness probabilistic scatter plot in CPS ≥5 group: **(A)** China, **(B)** The U.S.

**Figure 17:**
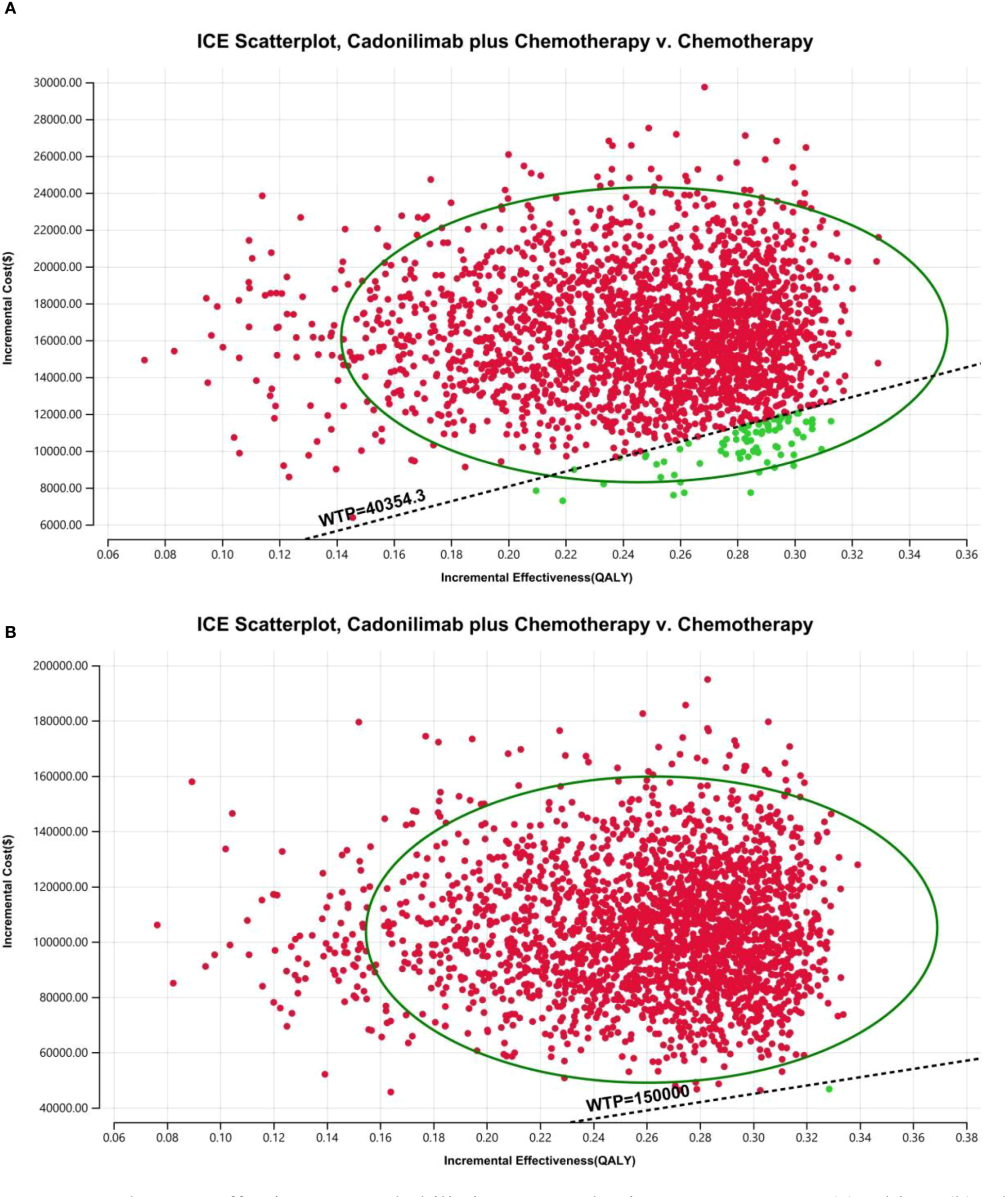
The cost-effectiveness probabilistic scatter plot in CPS <5 group: **(A)** China, **(B)** The U.S.

The PSA findings are shown in **Figures 12**–**17**. In a Chinese setting, the probability that cadonilimab plus chemotherapy would be cost-effective at the defined WTP threshold was 23.35% for the overall cohort, 64.37% for the CPS ≥5 subgroup, and 3.20% for the CPS <5 subgroup. In the U.S., the corresponding probabilities were 2.30% for the overall cohort, 1.48% for the CPS ≥5 subgroup, and 0.09% for the CPS <5 subgroup. These results further support the conclusion that cadonilimab plus chemotherapy offers limited economic value at the current price.

## Discussion

Cadonilimab, the first PD-1/CTLA-4 bispecific antibody approved for solid tumors, demonstrated notable clinical efficacy in improving both OS and PFS in patients with unresectable or metastatic GC/GEJC, as shown in the COMPASSION-15 trial. Results from a Bayesian network meta-analysis further supported its superiority: cadonilimab plus chemotherapy offered the greatest OS and PFS benefits among various ICI-based regimens, including nivolumab, pembrolizumab, sintilimab, tislelizumab, and sugemalimab, for HER2-negative GC/GEJC patients with positive PD-L1 CPS (36). This advancement marks a significant milestone in the era of bispecific antibodies for solid tumor immunotherapy and may reshape the global immunotherapy landscape.

Despite this clinical promise, our cost-effectiveness analysis revealed that cadonilimab plus chemotherapy is not economically viable as first-line treatment for most GC/GEJC patient groups in China under current or projected pricing. This conclusion was validated by scenario analyses: both PPP-adjusted currency conversion and a 3% discount rate (as a sensitivity check) confirmed the robustness of the base-case findings. Notably, however, the combination regimen was cost-effective in the PD-L1 CPS ≥5 subgroup—with an ICER of $37,499.27 per QALY, below China’s WTP threshold of $40,354.27. Corresponding INHB and INMB values were also positive, further supporting the use of cadonilimab in this clinically responsive population.

This subgroup-specific value is particularly relevant in the Chinese healthcare context, where cadonilimab has already undergone significant price reductions: following the 2024 national medical insurance negotiations, it was included in the 2025 national medical insurance catalog for cervical cancer (effective January 1, 2025). While GC/GEJC were not included in this round due to timing constraints, the rapid approval and price reduction of cadonilimab offer meaningful hope for GC/GEJC patients. Our findings provide strong evidence to support its future inclusion in medical insurance for GC/GEJC. This aligns with the broader landscape of ICIs’ cost-effectiveness analysis in China: nivolumab and pembrolizumab have been shown to be uneconomical (37–39), while the economic value of tislelizumab, sugemalimab, and sintilimab remains controversial (33, 40–44) —consistent with our results.

Conversely, in the United States, the ICERs were $290,498.45, $240,877.66, and $398,852.61 per QALY for the overall population, CPS ≥5, and CPS <5 groups, respectively—all well above the $150,000.00 WTP threshold. This is consistent with prior findings that pembrolizumab, nivolumab, and tislelizumab also lack economic viability in the U.S. GC/GEJC setting (39, 45–47). Notably, our ICER for cadonilimab is relatively closer to the U.S. WTP threshold than other ICIs, suggesting that further Network Meta-analyses or real-world studies may help clarify its comparative economic value.

Although cadonilimab has already undergone significant price reductions in China, its price remains undetermined in many other markets. Ongoing global trade tensions and tariff policies, particularly between China and the U.S., add to pricing uncertainty, potentially limiting access to high-value cancer therapies. Our price simulation provides important insights into pricing thresholds that could render cadonilimab cost-effective: below $1,047.71 per cycle in China and $4,958.77 per cycle in the U.S. Clinically, given its demonstrated safety and efficacy advantages (and lack of obvious economic disadvantages in select subgroups), we recommend that physicians tailor treatment plans to patients’ disease profiles (e.g., PD-L1 status) and financial capacities—prioritizing the most effective regimens when affordable. These findings can inform health insurance reimbursement adjustments and guide drug tiering in clinical practice guidelines.

Sensitivity analyses identified the cost of cadonilimab and utility values for PFS and PD as the most influential parameters on the ICER, highlighting the critical role of drug pricing and patient quality of life in determining economic value. The very low cost-effectiveness probabilities observed in the PSA further validated the robustness of our base-case conclusions.

To our knowledge, this study is the first to assess the cost-effectiveness of cadonilimab, a second-generation PD-1 inhibitor, combined with chemotherapy as a first-line therapy for GC/GEJC from both the U.S. and Chinese payer perspectives. Importantly, key model parameters (e.g., utility values, costs of best supportive care, and end-of-life care) were derived from GC/GEJC-specific studies to minimize input uncertainty.

Nonetheless, this study has several limitations that must be acknowledged. First, the clinical trial data used for modeling were derived exclusively from a Chinese population, which may limit their applicability to U.S. healthcare systems. Second, the model was based on data from a controlled clinical trial, which introduced inherent uncertainty. Although real-world patients often receive multiple lines of therapy, our model incorporates only up to second-line treatment, potentially introducing bias. Lastly, subsequent treatment proportions were reported at the single-agent level, which may have affected the accuracy of the post-progression cost estimates. Future studies that incorporate broader real-world data are required to validate and refine these findings.

## Conclusions

In China, cadonilimab combined with chemotherapy was cost-effective in patients with PD-L1 CPS ≥5, with an ICER of $37,499.27 per QALY—below the national WTP threshold of $40,354.27 per QALY. However, at current or projected prices, the therapy exceeded the WTP thresholds for all other subgroups in both China and the United States. These findings highlight the need to align clinical innovations with economic value to inform rational and equitable oncological treatment decisions.

## Data Availability

The raw data supporting the conclusions of this article will be made available by the authors, without undue reservation.
