# Supplementary material for: Cost-effectiveness of cadonilimab plus chemotherapy vs chemotherapy alone for advanced gastric cancer: evidence to inform drug pricing in the U.S. and China
Source: Front Immunol. 2025 Oct 14;16:1618726. doi: 10.3389/fimmu.2025.1618726 (PMC12560798; doi:10.3389/fimmu.2025.1618726)
Supplement: Supplementary file 1 [file DataSheet1.doc]

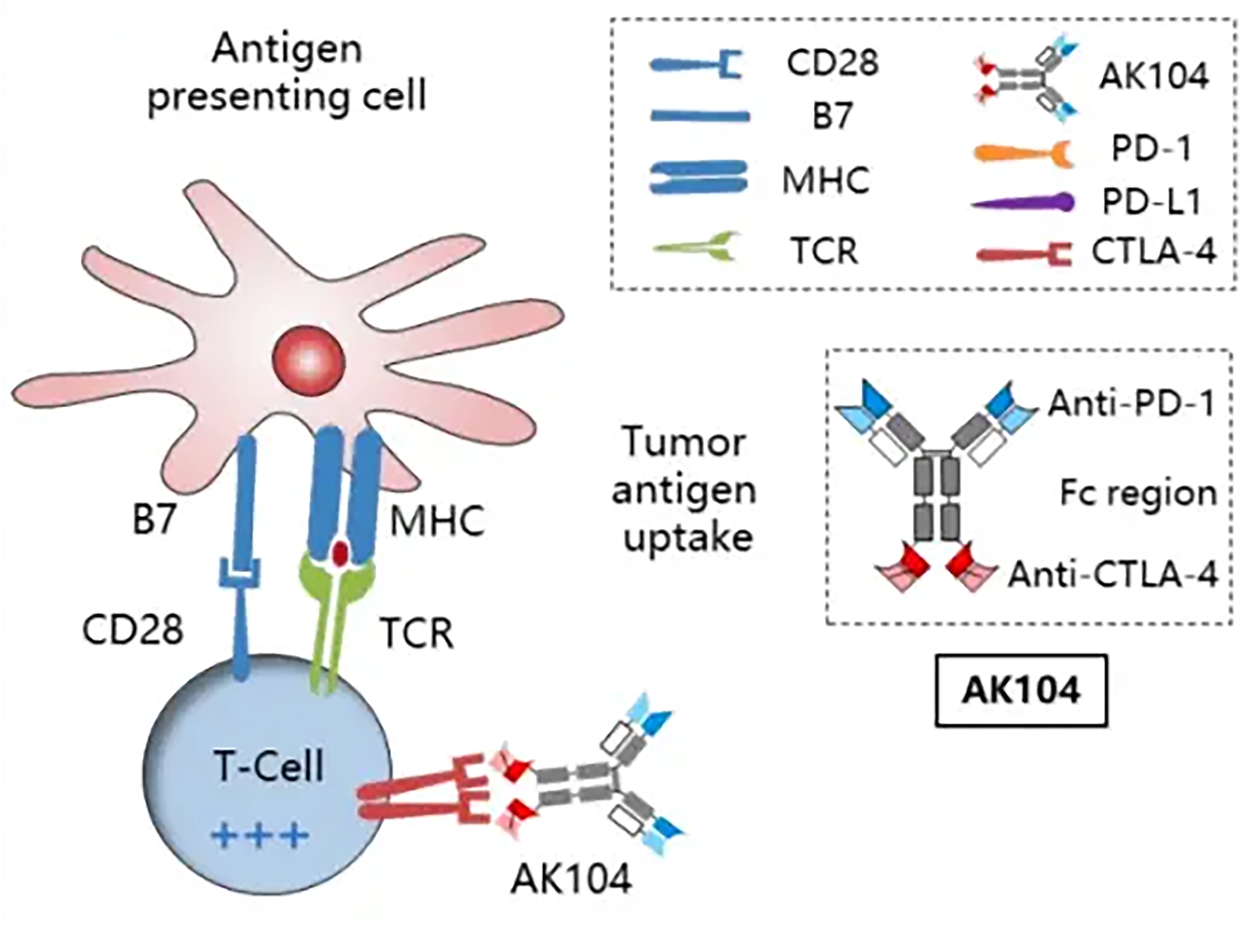


**Supplemental Figure 1.** Schematic diagram of cadonilimab tetravalent structure.

CD28：Cluster of Differentiation 28; B7：B7 Family Molecules; TCR：T Cell Receptor; MHC：Major Histocompatibility Complex; AK104：Cadonilimab; PD-1：Anti-programmed cell death protein-1; PD-L1：Programmed death ligand 1; CTLA-4：T-lymphocyte-associated protein 4.
